# Implementation of a ketamine programme for treatment-resistant depression in the public health system: Lessons from the first Australian public hospital clinic

**DOI:** 10.1177/00048674241237094

**Published:** 2024-03-18

**Authors:** Nicollette LR Thornton, Dean J Wright, Nick Glozier

**Affiliations:** 1 Central Clinical School, Faculty of Medicine and Health, The University of Sydney, Sydney, NSW, Australia; 2 ARC Centre of Excellence for Children and Families over the Life Course, Sydney, NSW, Australia; 3 Marie Bashir Centre, Royal Prince Alfred Hospital, Camperdown, NSW, Australia

**Keywords:** Ketamine, public hospital, implementation

## Abstract

One could argue that we are living through a period of innovation and change in psychiatry unlike that seen before, with repurposed medications emerging as novel treatments. However, despite evidence of enhanced clinical outcomes and potential medium-term savings, delivering these promising interventions is resource-intensive and perceived as difficult in the public sector. Consequently, they are generally only available in the private sector, often at great cost, effectively making them inaccessible to the ‘Have Nots’. The arrival of these paradigm-shifting treatments has inadvertently highlighted a growing mental health inequity. The Royal Prince Alfred Hospital’s Ketamine Treatment Clinic was the first public-sector ketamine treatment clinic for complex mood disorders in Australia. Based on 3 years’ experience establishing, developing and running a public-sector ketamine treatment service, we review the progress, perils and pitfalls for clinicians and health services contemplating establishing a public-sector ketamine treatment service of their own.

## Introduction

Treatment-resistant depression (TRD), commonly defined as non-response to two or more antidepressants of adequate dose and duration in the current episode of depression, is often associated with chronic vocational dysfunction and benefit dependency (Johnston et al., 2019; Taipale et al., 2020). It is more prevalent in lower socioeconomic groups who frequently do not have health insurance (Al-Harbi, 2012). The novel treatments for TRD (Transcranial Magnetic Stimulation, ketamine and psychedelic-assisted psychotherapy) are expensive and generally only available in private settings. This health inequity would be intolerable in most other specialities in Australia and New Zealand, where lack of finances may lead to slower access rather than a frank denial of access to treatment. If we are to treat the majority with TRD, we need to provide these treatments in the public sector.

Public psychiatric services have traditionally focussed on ‘serious’ mental illnesses such as psychoses, with chronic depressive illnesses often managed in either private psychiatry or, more commonly, primary care, with intermittent admissions for acute self-harm or suicidal risk. TRD is challenging to manage, often cycling through different antidepressant classes, adjunctive medications, and electroconvulsive therapy. Many with TRD have few easily accessible, publicly funded treatment options.

One potential avenue for this population is ketamine treatment. Ketamine services are increasingly available in Australia, particularly with the recent Therapeutic Goods Administration (TGA) approval of intranasal esketamine (Spravato®; Janssen-Cilag Pty Ltd). Ketamine treatments are often self-funded, are ineligible for health insurance rebates and available only through private providers at substantial cost. The Royal Prince Alfred Hospital Ketamine Treatment Clinic in Sydney was the first public ketamine treatment service in Australia and provides ketamine treatments at no cost to the patient. The initial implementation in 2021, subsequent scaling up of the service and the ensuing experience operating our clinic form the basis for our recommendations to those planning to implement their own public clinics.

There are many considerations common to both public and private settings. These include dosing protocols, safety monitoring, management of side effects and regulatory issues related to prescribing Schedule 8 (Australia)/Class C (New Zealand) drugs (Bayes et al., 2019, 2021; Ceban et al., 2021; McIntyre et al., 2021; Short et al., 2020; Thornton et al., 2023a). A major concern for anyone setting up a ketamine treatment service is that treatments are resource-intensive, and a significant component of the resource burden relates to safety monitoring. Patients must be administered the treatment in an appropriate clinical setting, under supervision, and following treatment administration should be monitored for approximately 2 hours, until clinically stable and suitable for discharge. This *Viewpoint* focusses on concerns germane to the public sector.

## Clinical pathways

### Referrals and responsibility

Our public model does not have the resources for a full mood disorder service, as often seen in the private sector, instead offering ketamine as an adjunctive treatment. Many patients referred to our clinic are not managed by a private psychiatrist and/or psychologist, often due to financial or access constraints. By necessity, we accept referrals directly from General Practitioners. Others may be managed by community or specialist teams (e.g. eating disorder teams), and decisions regarding who is the main treating specialist need to be clear. Thus, additional administrative support is often required: Liaising with treating doctors regarding necessary medication changes; preventing changes that can complicate treatment; and liaising with psychologists about approaches during treatment. This creates collaborative relationships with primary care and colleagues, fostering continuity post-discharge.

### Standardising processes

In an environment where funding is limited, it is important to develop clear guidelines relating to inclusion, assessing benefit, treatment duration, timely discharge for patients who do not benefit and discharge planning for those who do. Guidelines further inform training requirements and facilitate communication with patients and referrers.

We adapted inclusion criteria from recent clinical trials. However, we built in more flexibility around suicidal ideation and self-harm, past substance use and comorbidities (the norm in our population). We retained the requirement that patients meet the clinical criteria for TRD. Although there are many definitions, we require people display non-response to ⩾2 antidepressants of adequate dose and duration, in the current episode of depression (McIntyre et al., 2023).

All patients accepted into the programme are treated for a minimum of 4 weeks of twice-weekly treatment. In practice, we find this gives sufficient time to assess whether a patient will benefit from treatment, as typically we see gradual, rather than sudden symptom improvements. For treatment responders, this is followed by a further 4 weeks of reducing frequency before a period of maintenance treatment focussed on functional gains. Despite the claims, we rarely see such gains in the initial phase in our disadvantaged population. The structured approach facilitates management of patient expectations by giving clarity around timelines and minimises use of resources for patients who receive no therapeutic benefit.

### Treatment outcomes

In line with recommendations, we use a measurement-based care model based on the standardised scales utilised in clinical trials (Bayes et al., 2021). The scales we use are embedded into a clinical quality network to ensure ongoing evaluation of best practice (Clinical Alliance and Research in ECT & Related Treatments Network [CARE Network]; Martin et al., 2018). We use the Montgomery Åsberg Depression Rating Scale (MADRS) as the primary measure of treatment response (⩾50% reduction compared to pre-treatment baseline), which forms the basis of each clinical assessment. In instances where a patient has not displayed a ⩾50% reduction in the MADRS but seems to display therapeutic benefits, secondary scales can be used to inform whether a further trial of treatment can be justified. We collect freely available patient-report measures that can be easily administered to patients, covering depression, anxiety, sleep, functional disability and quality of life, among others. While some patients dislike clinical measurements, most seem to value the process of self-reflection and visualising change. For us, the extra time required is essential as we attempt to justify funding for this novel treatment in the public sector.

In the public system, with chronically unwell, functionally impaired patients, we have reframed what are considered ‘reasonable expectations’ for therapeutic outcomes and recovery. In the patients we see with multiple comorbidities, a lengthy history of non-response to treatments, social isolation and often years on the Disability Support Pension or National Disability Insurance Scheme, should a good outcome be characterised as remission, better quality of life or some other functional aspect? Is sustained clinical response more reasonable and realistic?

### Treatment duration and re-referrals

The ethical approach differs in the public sector, as clinicians must balance individual patient needs while maximising benefit to the community. We do not have the ‘elastic’ capacity of the private sector. The rapid-acting antidepressant effect of single-dose ketamine often fades within a week and thus, repeat-dosing is deemed necessary to prolong the acute efficacy (Kryst et al., 2020; Xu et al., 2016). Discharging patients who have benefitted from treatment is challenging, as those who remit often fear relapse. Many patients do well without ongoing treatment, avoid relapse and require no subsequent episodes of care. To date, we have discharged 31 patients from our clinic who have met either response or remission criteria and only 4 of these have been re-referred.

For other responders, continued treatment may not lead to remission. Our experience suggests this is more commonly the case. Such patients will often end up in a holding pattern, relying on ongoing ketamine treatment to maintain partial benefits. Unfortunately, this means our waitlist continues to grow and many patients who cannot afford private treatment do not receive access to ketamine.

Clinicians should consider carefully how they manage patients who have responded but not remitted. Many tell us this is as good as they have been for years despite not remitting. However, we need throughput so we can treat new referrals, and must make decisions often unpalatable to patients, especially when many have become very engaged with the clinic.

In February 2023, a meeting was convened by the Pharmaceutical Benefits Advisory Committee (PBAC) during their evaluation of Spravato’s proposed Pharmaceutical Benefits Scheme (PBS) inclusion. Clinician-experts opined that it would be appropriate in the majority of cases to limit treatment with either ketamine or esketamine to 12 months, noting that ‘some individuals’ may require lengthier treatment, and some may re-present for treatment later (Department of Health and Aged Care, 2023a). This consensus has been useful for us to benchmark a standard treatment duration.

With time we have been faced with discharged treatment-responders being re-referred for treatment. Should re-referred patients be required to meet the criteria for TRD in the new episode before being eligible for further treatment? Alternatively, does their history of treatment non-response mean they do not need to trial further medications prior to being re-referred? We have a waitlist for treatment: should known responders jump the queue?

### Ketamine treatment strategies

Engagement in behavioural modification during the acute period of post-treatment neural plasticity may enable stronger, long-term therapeutic outcomes. This is taken from clinician-observations and patient-reports collected for a research project, and aligns with emerging data on the use of positive environmental modification to enhance the benefit of ketamine (Duman et al., 2016; Price et al., 2022). Modifications commonly include promoting exercise, reengaging in paid/volunteer work, study, hobbies, social activities and psychotherapy. Many patients have no or limited access to psychotherapy, so treatment providers should consider whether there are subsidised or other pathways available, e.g., the psychotherapy training programme for psychiatry registrars or clinical psychologists.

## Funding treatment

Public health services in Australia are funded via two pathways: an ‘activity-based funding’ (ABF) model, where funding is based entirely on the number of patients seen and the type of service provided, and a ‘block funding’ model of fixed-plus-variable funding where the variable component is based on activity.

The primary expense involved in operating our clinic is staff wages, rather than medication costs as has previously been raised as a major issue by Rodgers et al. (2024). For day-to-day operations, the minimum staff we require is a psychiatry registrar (under the supervision of a consultant who also has to do much of the associated administration work with S8s, etc.) and a registered nurse. Their respective expertise is necessary, and two clinical staff are required to comply with Schedule 8/Class C handling requirements. Other staff may be needed based on the scale of the service. In addition to these, we have a clinical psychologist and utilise volunteers as administrative staff members. Without a pre-existing business case, and for a group that appear not to be seen as core business of public secondary mental health services, we needed to provide ‘start-up’ funds from other sources until feasibility and outcomes had been demonstrated to the organisation. Openness to change from an institution can make-or-break the translation of evidence into practice (Correa et al., 2020). While we had in-principle support from management, we did not have complete buy-in, and this has resulted in challenges with sustainability of the clinic. For example, in the establishment phase and the initial 12 months of operations, our clinic was partly funded by clinical trials income and Medicare billing, rather than service funds. Increased efforts from us to gain full management engagement in the early stages may have avoided this issue.

The number of patients treated per clinic is more heavily contingent on space than staff availability. We do not use beds for each patient, increasing capacity by providing recliner chairs. It is likely there is existing infrastructure that can be adapted for the purposes of providing treatment. We found the ECT suite is ideal as it has appropriate drug storage facilities, blood pressure monitoring and emergency equipment, and is only used part-time. Other spaces such as community mental health facilities and long-acting olanzapine clinics could be adapted.

With a minimum wage expenditure as well as other fixed costs, regardless of the funding model, a funding threshold must be met. In developing a business case under an ABF model, a benchmark number of patients and activity per clinic can be calculated. For a block-funded service, a minimum investment would be required from the service, regardless of the number treated. In either case, there needs to be careful identification of roles, service and treatment model (e.g. infusion and subcutaneous injection takes the same monitoring time but receives different ABF funding).

## Treating in inpatient settings

An under-researched area is ketamine’s role in treating inpatients, potentially reducing admission duration – a major cost-saving opportunity if established. To our knowledge, there has been no systematic evaluation of the effect of inpatient treatment on duration of admissions. However, a retrospective chart analysis of 37 patients in a private clinic suggested a 70% reduction in inpatient hospital days and 65% reduction in admissions following oral ketamine treatment compared to the equivalent period prior to treatment initiation (Hartberg et al., 2018).

In practice, we found the logistics of providing inpatient ketamine treatment to be complex. Should inpatients be treated on a ward, or in a specialty outpatient facility, with specifically trained staff and a contained environment? The model of treating inpatients in ECT suites would seem ideal but raises concerns about patient transfers. Who is responsible for providing nursing resources for one-to-one observation and how does the service accommodate that? Conversely, if ketamine is provided on the ward, appropriate training must be provided to large numbers of rotating ward staff who will provide care on treatment days. Discharge from the inpatient setting and potential for outpatient ketamine treatment must be managed between the inpatient and outpatient teams.

One concern from managers was the possibility of individuals presenting to emergency services for the purposes of accessing treatment via an admission. There have been anecdotal reports of increased demand for access to clinical trials of psychedelic treatments for mental ill-health in response to optimistic media coverage of psychedelics (Kent, 2022; Noorani, 2020). Our previous research has demonstrated that ketamine treatments are portrayed by the media in an overly optimistic light (Thornton et al., 2023b). In an already overburdened system, unnecessary presentations for the explicit purpose of accessing ketamine is an understandable, though theoretical, concern.

## Training

In the RANZCP guidelines for the use of ketamine in practice, psychiatrists with experience administering ketamine should manage treatment, effectively requiring any new provider to have trained elsewhere (The Royal Australian & New Zealand College of Psychiatrists, 2022). To our knowledge, the only formal training currently available in Australia is a 2-day RANZCP-endorsed course offered by The Black Dog Institute in Sydney. In the public sector, trainee psychiatrists play a major role in providing clinical care. At present, ketamine and other novel therapies such as psilocybin- and MDMA-assisted psychotherapy are not incorporated into the RANZCP registrar training programme. Future psychiatrists need to be given exposure to, under appropriate supervision, treating patients with these novel therapies. Although there remains a lack of consensus for optimum treatment protocols, incorporating ketamine into the formal training programme, with input from experts and the college around the way these training services operate, would help ensure the future workforce are trained to the highest standards.

At our clinic, we have set up a clinical and research registry, linked with the larger CARE Network. Information is collected as part of routine care and, with appropriate approvals, can be used for research purposes. A goal of our registry was to increase research capacity, facilitating research training opportunities for registrars to complete their scholarly projects. By building the registry into the clinic model, we can foster the development of core research skills, without the effort and timelines involved in collecting data in real time.

## Public-sector pharmacy provision and medication governance

The choice of formulation is an important consideration for prescribers. Of the two available on the market, the first – racemic ketamine – has been used clinically for approximately 50 years and is TGA approved for use in sedation and anaesthesia. It is readily available and may cost a hospital as little as $5–$10 for a subcutaneous, intravenous or oral dose. Work by Loo et al. (2016) compared different routes that racemic ketamine can be administered, demonstrating subcutaneous injections were well tolerated and associated with fewer adverse events compared to intravenous and intramuscular routes. The equipment and expertise needed to administer subcutaneous injections is also less specialised than intravenous infusions, but the evidence base for the latter is greater. The off-label prescription of racemic ketamine may pose challenges for the prescriber, depending on institutional policies and procedures, and health jurisdiction-based approval processes. The pharmacy branch of NSW Health has worked with clinicians to establish an approval process for ongoing treatment with racemic ketamine, requiring a second opinion from a psychiatrist. However, prior to accessing our clinic, many of our patients were unable to access even a ‘first opinion’.

The second available formulation – intranasal esketamine (Spravato®) – is TGA approved for use in TRD when co-initiated with a new oral antidepressant (Janssen-Cilag Pty Ltd, 2022). In August 2023, the PBAC again rejected an application to list the drug on the PBS (Department of Health and Aged Care, 2023b). The obvious consequence is that, outside of industry-sponsored compassionate programmes, or health insurance funding, clinicians wishing to treat their patients with esketamine must ask patients to fund the full drug cost or make applications for the hospital to fund it at up to approximately $800 per dose. In the current circumstances, it may be difficult to justify the use of esketamine to a local Drug and Therapeutics Committee when racemic ketamine is readily available at a fraction of the cost, but this may change.

Other preparations of ketamine have been trialled (e.g. extended-release tablets; Glue et al., 2020) with promising results (Douglas Pharmaceuticals, 2021). If, and when new drugs become approved, clinicians will be faced with the same quandary as with esketamine: prescribe a more affordable formulation off-label, or an on-label formulation for which the patient or the hospital foots the – likely substantial – bill. There is no guarantee that the PBAC will not form the same views of these emerging preparations as they have of esketamine.

## Summary

While this endeavour has been challenging, establishing this clinic has been extremely rewarding. It would be disingenuous to suggest that establishing a public ketamine treatment service is straightforward and did not require substantial personal investment. However, we have demonstrated it is feasible and fulfilled an unmet need at our health service. There are many challenges unique to a public health setting that must be carefully considered and addressed if ketamine treatment is to become more widely available. These challenges are multilevel from the individual clinician to the health service, and to government policy. They are not easy to address, but overcoming them would significantly contribute to reducing the barriers to accessing affordable and effective treatments that those with TRD face.
